# A Retrospective Review Exploring the Role of Gender and Frequency of Tonsillectomy, Adenoidectomy, and Tympanostomy in Pediatric Patients With Speech Delay

**DOI:** 10.7759/cureus.102826

**Published:** 2026-02-02

**Authors:** Raj Patel, Steven Rua, Janaki Patel, Germaine Harvey, Rebecca Maddrell

**Affiliations:** 1 Otolaryngology-Head and Neck Surgery, Loyola University Chicago Stritch School of Medicine, Maywood, USA; 2 Family Medicine, Loyola University Medical Center, Maywood, USA

**Keywords:** adenotonsillectomy, demographic features, gender representation, speech and language delay, tympanostomy tube

## Abstract

Background

Speech and language delays are common early childhood conditions that involve difficulty with sound formation and language comprehension. Factors that often contribute to these delays include hearing loss, oral impairments, and frequent ear and throat infections, among others. Otitis media is a common pediatric cause of conductive hearing loss, which can lead to speech delays, and is often treated with tympanostomy tubes. Sleep apnea, often caused by enlarged tonsils and adenoids in children, can also affect speech by disrupting sleep and thus cognitive development. Tonsillectomy and adenoidectomy can help restore proper airflow and sleep quality, supporting learning and speech development. This study examines the prevalence of these procedures in children with speech delay and their relationship to demographic factors such as gender, ethnicity, and socioeconomic status.

Methods

A retrospective case-control chart review was conducted to identify pediatric patients from birth to five years diagnosed with speech delay, who underwent tympanostomy, tonsillectomy, and/or adenoidectomy from May 2020 to September 2025. Patients diagnosed with speech delay were identified using the F80.9 ICD diagnosis code for developmental disorders of speech and language. Patients who underwent these procedures were identified by their respective procedure codes: tympanostomy (69436), tonsillectomy (42825/42826), and adenoidectomy (42830/42831). Demographic information relating to gender, race, and income was also collected. Statistical analysis was done in Research Electronic Data Capture (REDCap) (Vanderbilt University, Nashville, Tennessee, United States) using chi-squared and regression analysis.

Results

Of 885 pediatric patients with speech delay, 63.2% underwent at least one of the listed surgeries, with 71.8% being male and 49.4% female (p<0.01). Regression analysis showed males were 1.84 times more likely to undergo surgery than their female counterparts (p<0.001). Sixty percent of patients who received surgery underwent more than one procedure, with tympanostomy being the most common (37.4%). Caucasians had the highest rate of procedures (38.1%; p<0.01), and most patients were from middle- and lower-income households (p<0.01).

Conclusion

Male pediatric patients with speech delay were significantly more likely to undergo tympanostomy, tonsillectomy, and/or adenoidectomy than females. Most patients underwent more than one procedure, reflecting common comorbid conditions and, therefore, the need for combined intervention. Caucasian ethnicity and middle to lower socioeconomic status were also associated with higher procedure rates.

## Introduction

Speech development is a crucial process by which children learn to produce sounds and form words and phrases to communicate effectively [1]. This critical development primarily occurs by age three. By 10 months to two years old, children typically expand their vocabulary to 50 words and begin to combine two words into simple phrases or short sentences. Speech delay is often identified when a child's speech and language development does not progress at the expected rate for their age. This condition is most commonly diagnosed in children under two years old. The literature indicates that 15-20% of children exhibit speech delay by age two, while 5-8% of children aged 3-10 have speech delay, highlighting the greater prevalence of speech delay among younger pediatric patients [2,3].

Well-known risk factors for speech delay include male gender, family history of speech and language disorders, low birth weight, prematurity, and lower socioeconomic status. The etiology of speech delay is broad, encompassing conditions such as hearing loss, oral impairments, autism, intellectual disability, cerebral palsy, otitis media, tonsillitis, adenoiditis, and obstructive sleep apnea (OSA) [4]. Among these, ear and throat infections have been shown to significantly exacerbate speech delay. Additionally, though sleep apnea is not the most common cause of speech delay, it is recognized as a contributing factor [5].

Incidentally, the critical age for speech development, around 2-3 years, is also when children are most susceptible to infection. In the case of otitis media, chronic middle ear effusion can lead to conductive hearing loss, which may interfere with a child's ability to naturally absorb language by hearing spoken words. Similarly, throat infections such as tonsillitis and adenoiditis often are accompanied by pain and difficulty speaking, which may hinder vocalization and projection. These factors contribute to the worsening of speech delays, making early intervention crucial in managing the impact of such infections [5,6]. Around 80% of children will experience an episode of otitis media, with 6-24-month-olds being the most common age group for these infections [7]. Approximately 61% of children will be affected by tonsillitis and/or adenoiditis, with the prevalence increasing as children get older [8]. These statistics highlight the significant role that ear and throat infections play in speech development and emphasize the importance of addressing them promptly. 

In addition to infection, OSA is linked with neurocognitive issues due to fragmented sleep and chronic hypoxia. These disruptions impair brain development, particularly in regions responsible for language, memory, and executive function, and have the potential to result in speech delay. There is currently no literature that quantifies the percentage of pediatric patients with OSA who develop speech delay. However, the association between these conditions is clinically recognized. The prevalence of OSA in otherwise healthy pediatric patients ranges from 1% to 5%. OSA is most common in patients between the ages of two and three years, primarily due to adenotonsillar hypertrophy, which tends to peak during this period. It is important to note that the prevalence of OSA can reach 20-30% or higher in pediatric patients with risk factors such as increased neck circumference, overweight or obesity, and craniofacial abnormalities [9,10].

Tympanostomy tube insertion is an effective intervention for managing recurrent otitis media with effusion. By draining the fluid and equalizing pressure, tympanostomy tubes restore proper sound conduction, thereby supporting normal hearing and facilitating speech acquisition. Further, tonsillectomy and adenoidectomy work to reduce the volume of tissue contributing to obstruction in OSA, thereby increasing airway patency and improving sleep quality and oxygenation [9,10].

The purpose of this paper is to determine the prevalence of tympanostomy tube placement, tonsillectomy, and adenoidectomy in pediatric patients with speech delay and to identify the rate at which these procedures are performed in this population. Additionally, demographic information such as gender and socioeconomic status will be analyzed to better understand the profiles of these patients.

## Materials and methods

Study design and institutional approval

This study was designed as a retrospective chart review. Approval for study conduct and authorization to access and utilize the Loyola University Medical Center electronic medical record (EMR) system were obtained prior to data collection through the institutional review process. The study was conducted in accordance with institutional policies and ethical standards for retrospective human subjects research.

Data source

Patient data were extracted from the Loyola University Medical Center EMR system which serves as the primary clinical documentation and data repository for inpatient and outpatient encounters within the health system. Permission to use the EMR for research purposes was granted as part of institutional approval. Extracted data were subsequently managed and stored using REDCap (Research Electronic Data Capture) (Vanderbilt University, Nashville, Tennessee, United States), a secure, web-based application designed for research data management.

Study population

Pediatric patients diagnosed with speech delay were identified using the International Classification of Diseases, Tenth Revision (ICD-10) diagnostic code F80.9 (developmental disorder of speech and language, unspecified). Patients were included if they had a documented diagnosis of speech delay within the study period. 

Procedural history was assessed using Current Procedural Terminology (CPT) codes to identify relevant otolaryngologic interventions. Tympanostomy tube insertion was identified using CPT code 69436. Tonsillectomy was identified using CPT codes 42825 and 42826, while adenoidectomy was identified using CPT codes 42830 and 42831. 

Inclusion criteria consisted of pediatric patients who were diagnosed with speech delay anytime from zero to five months of age. Any patient older than five months and/or who was not diagnosed with speech delay was excluded. 

Variables and data collection

Demographic variables collected included patient age, sex, gender, ethnicity, and residential zip code. Surgical variables included history of tympanostomy tube insertion, tonsillectomy, adenoidectomy, or combinations thereof.

Socioeconomic status was estimated using zip code-level adjusted gross income (AGI) data. Patient zip codes were matched to AGI data to estimate median household income. Income levels were stratified into three socioeconomic classes: lower class, middle class, and upper class. Median income values were calculated for each income class.

Study period

Chart review encompassed patient encounters documented between May 2020 and September 2025. 

Statistical analysis

Descriptive statistics were used to summarize demographic and clinical characteristics. Chi-squared tests of independence were applied to assess associations between categorical variables, including sex and surgical intervention status. Regression analysis was performed to evaluate relationships between demographic, socioeconomic, and procedural variables and patient outcomes. Statistical significance was defined using standard thresholds. All analyses were conducted using compiled datasets generated from REDCap. All statistical analyses were performed using R Version 4.5.2 (R Foundation for Statistical Computing, Vienna, Austria). Chi-squared tests of independence and logistic regression analyses were conducted, and a p-value of <0.05 was considered statistically significant.

## Results

A total of 885 pediatric patients were diagnosed with speech delay between May 2024 and September 2024. Furthermore, 63.2% (559/885) of patients underwent at least one procedure, while 36.8% (326/885) did not undergo any (see Table 1). Of those diagnosed with speech delay, 71.9% (636/885) were male, while 28.1% (249/885) were female (p<0.01). Among males, 49.4% (437/885) underwent at least one procedure, compared to 13.8% (122/885) of females (p<0.01). The statistical significance of these findings suggests a strong association between gender and the likelihood of undergoing tympanostomy tube placement, tonsillectomy, and/or adenoidectomy. This association is further supported by a Cramér's V of 0.61, indicating a moderate to strong relationship between gender and procedure frequency (see Table 2). 

**Table 1:** Procedure prevalence Statistical test: chi-squared test of independence df=1; χ²≈64; p<0.01

Patient type	Received procedures (%)	Did not receive procedures (%)	Total	P-value
Speech delay patients	559 (63.2%)	326 (36.8%)	885	p<0.01

**Table 2:** Gender distribution Statistical test: chi-squared test of independence df=1; χ²≈29.8; p<0.01 Cramér's V=0.61

Gender	Received procedures (%)	Did not receive procedures (%)	Total	P-value
Male	437 (49.4%)	199 (22.5%)	636 (71.9%)	-
Female	122 (13.8%)	127 (14.4%)	249 (28.1%)	
Total	559 (63.2%)	326 (36.9%)	885	p<0.01

Next, a regression analysis revealed that males with speech delay were 1.84 times more likely to undergo at least one procedure compared to their female counterparts (see Table 3). The coefficient for males (0.6104) is highly significant (t-statistic=24.87; p<0.001), with a 95% confidence interval of 0.5623-0.6586. The coefficient for females (0.1695) is also significant but smaller (t-statistic=5.49; p<0.001), with a confidence interval of 0.1089-0.2302. These results emphasize the significant role of male gender in the increased likelihood of receiving these procedures in pediatric speech delay cases.

**Table 3:** Regression analysis of gender Statistical test: linear regression model

	Coefficients	Standard error	t-statistic	P-value	Lower 95%	Upper 95%
Male	0.61042945	0.02454769	24.8670795	6.964E-104	0.56225081	0.65860808
Female	0.16953477	0.03088705	5.48886269	5.2878E-08	0.10891418	0.23015537

Around 60.1% (336/559) of patients were categorized as having received multiple procedures, indicating that the majority of those with speech delay underwent more than one type of intervention, such as tympanostomy tube placement, tonsillectomy, or adenoidectomy. This includes procedures performed either during the same operation or at different times (p<0.01). The second most common procedure was tympanostomy tube placement, which accounted for 37.4% (209/559) of all procedures (see Table 4). 

**Table 4:** Surgery type distribution Statistical test: chi-squared test of independence df=3; χ²≈563; p<0.01

Surgery type	Count (%)	P-value
Multiple surgeries (tonsillectomy/adenoidectomy, tympanostomy/tonsillectomy/adenoidectomy)	336 (60.1%)	-
Tympanostomy	209 (37.4%)	
Adenoidectomy	13 (2.32%)	
Tonsillectomy	1 (0.17%)	
Total	559	p<0.01

Of the 885 patients diagnosed with speech delay, 50.1% (443/885) were Caucasian and 23.9% (212/855) were African American (p<0.01) (see Table 5). Among patients who underwent surgery, 34.7% (307/885) were Caucasian (p<0.01). These findings highlight a statistically significant association between ethnicity, the diagnosis of speech delay, and the prevalence of surgery within these groups. 

**Table 5:** Ethnicity distribution Statistical test: chi-squared test of independence df=5; χ²≈50; p<0.01

Ethnicity	Received procedures (%)	Did not receive procedures (%)	Total (%)	P-value
Caucasian	307 (34.7%)	136 (15.4%)	443 (50.1%)	-
African American	139 (15.7%)	73 (8.24%)	212 (23.9%)	
Asian	6 (0.68%)	9 (1%)	15 (1.69%)	
Hispanic	71 (8%)	59 (6.7%)	130 (14.7%)	
Native American	1 (0.11%)	2 (0.22%)	3 (0.34%)	
Other	35 (3.9%)	47 (5.3%)	82 (9.27%)	
Total	559 (63.2%)	326 (36.8%)	885	p<0.01

Lastly, 58.8% (520/885) of patients were from the middle class, 29.5% (261/885) from the lower class, and 11.8% (104/885) from the upper class (p<0.01). When stratifying the socioeconomic cohorts into whether procedures were done or not, the middle class and lower class had a higher predominance of 34.2% (303/885) and 21.8% (193/885), respectively (see Table 6).

**Table 6:** Socioeconomic class distribution Statistical test: chi-squared test of independence df=2; χ²≈18.4; p<0.01

Socioeconomic class	Procedures received (%)	Did not receive procedures (%)	Total (%)	P-value
Higher class (>$120k)	63 (7.1%)	41 (4.6%)	104 (11.8%)	-
Middle class ($60k-120k)	303 (34.2%)	217 (24.5%)	520 (58.8%)	
Lower class (less than $60k)	193 (21.8%)	68 (7.7%)	261 (29.5%)	
Total	559 (63.2%)	326 (36.8%)	885	p<0.01

## Discussion

Speech delay is a relatively common childhood condition, affecting approximately 7-10% of children in the United States. Speech delay encompasses a broad range of etiologies; however, ear and throat infections are found in nearly 60% of affected patients [11]. These infections can lead to temporary or chronic hearing loss, which directly impacts language acquisition and speech development. Recurrent otitis media, for example, may cause fluctuating conductive hearing loss, disrupting a child's ability to perceive sounds accurately during critical periods of speech and language learning [12]. Additionally, chronic throat infections and associated inflammation can contribute to discomfort or structural changes that affect articulation and vocal quality. It is therefore essential to consider both the direct and indirect effects of these infections when evaluating and managing speech delays in pediatric populations [13]. Early identification and treatment of ear and throat conditions may play a key role in improving speech outcomes.

Additionally, OSA leads to disrupted sleep architecture and intermittent hypoxia, both of which can adversely affect cognitive function, behavior, and overall neurodevelopment [10]. Studies have shown that children with untreated OSA are at increased risk for attention deficits, learning difficulties, and behavioral problems, which may further compound delays in speech and language development [9,10]. Moreover, the inflammation and upper airway obstruction associated with OSA can contribute to oral motor dysfunction, potentially impacting articulation and voice quality [10]. Early diagnosis and management, including adenotonsillectomy or continuous positive airway pressure (CPAP) therapy when indicated, are critical to mitigating these effects and improving long-term developmental outcomes [10].

Given that these conditions are major risk factors for speech delay, this study aimed to analyze the prevalence of tympanostomy tube placement, tonsillectomy, and adenoidectomy among patients with speech delay and to assess their demographic characteristics.** **It is important to understand how the development of these infections impacts the ability of a developing pediatric patient to progress in their speech.

Analysis of the patient cohort revealed that 63.2% of pediatric patients with speech delay underwent at least one procedure, with 60.1% of those patients receiving multiple interventions, such as tympanostomy tube placement with tonsillectomy or tonsillectomy with adenoidectomy (see Table 1 and Table 4). Tympanostomy tube placement alone was the second most common procedure, with a prevalence of 37.4% (Table 4). These findings provide critical insights into the relationship between speech delay and ear/throat infections. First, the high prevalence of these procedures highlights the significant role recurrent infections play in speech development and demonstrates the importance of surgical intervention in addressing infection-related speech delays. Second, the predominance of tympanostomy tube placement suggests that chronic otitis media may have a greater impact on speech development than tonsillitis and/or adenoiditis, emphasizing the crucial role of auditory input in speech acquisition. This aligns with the understanding that chronic otitis media can cause conductive hearing loss and a reduction in sound clarity, thus negatively affecting pronunciation, articulation, and overall speech intelligibility.

The prevalence of tympanostomy tube insertion in the United States for pediatric patients around two years of age is 25% [14]. Comparing this national percentage to the cohort of this study, which is 37.4%, it demonstrates a possible relationship whereby pediatric patients with speech delay have a higher need and prevalence of such procedures compared to pediatric patients who are not diagnosed with speech delay. It can also provide a possibility that patients with speech delay are more susceptible to ear infections than the average pediatric patient. Future research is required to address such observations and provide substantial evidence of these relationships. 

Another key finding of this study was the high prevalence of male pediatric patients with speech delay (see Table 2). Males comprised 71.8% of the speech delay cohort and 78.2% of those who underwent at least one procedure. This provides strong evidence that male gender is a risk factor for both speech delay and ear/throat infections. These findings are consistent with existing literature, which indicates that males are 2.5 times more likely to develop speech delay than females [15]. In this study, males with speech delay were 1.84 times more likely to undergo tympanostomy tube placement, tonsillectomy, and/or adenoidectomy. This data highlights the predominance of male patients being diagnosed with speech delay and the increased likelihood of requiring surgical intervention for associated infections [16]. There is little research on investigating the question of why males are more associated with speech delay and ear/throat infections. Possible explanations have been made that male hormones, genetic differences, and environmental factors may play a role in this observation [17]. 

Lastly, the higher prevalence of patients from middle and lower socioeconomic classes in the cohort (see Table 6) suggests a potential link between socioeconomic status, speech delay diagnosis, and the need for procedures to treat ear and throat infections. Existing research has established a correlation between lower socioeconomic backgrounds and developmental challenges in pediatric patients, including speech delays [17]. Additionally, children from lower socioeconomic backgrounds have a higher susceptibility to infections such as otitis media, often resulting in an increased need for tympanostomy tube placements compared to those from higher socioeconomic levels [17]. These disparities underscore the influence of social and environmental factors on speech development and overall health outcomes.

Limitations 

This study has several limitations. First, it is a retrospective chart review, which is inherently subject to selection bias and relies on the accuracy and completeness of EMRs. The use of ICD and CPT codes may not capture all patients with speech delay or procedures performed, potentially leading to underestimation or misclassification. Second, the study was conducted at a single institution, limiting the generalizability of the findings, as practice patterns and patient demographics may differ in other regions or healthcare systems. Socioeconomic status was estimated using zip code-level AGI, which may not accurately reflect the true income or resources of individual households, potentially introducing misclassification and bias in analyses examining income-related trends. Additionally, although regression analysis was used to evaluate associations, unmeasured confounders, such as comorbid medical conditions, parental preferences regarding surgical interventions, or provider-specific decision-making practices, could influence which patients underwent procedures, affecting the observed associations between demographic factors and surgical prevalence. Finally, the study period was limited to five months, which may not capture longer-term trends, seasonal variations, or changes in practice patterns over time. Despite these limitations, the findings provide meaningful insight into the demographic patterns and prevalence of surgical interventions in pediatric patients with speech delay.

## Conclusions

There is a significant association between the male gender and the likelihood of undergoing tympanostomy tube placement, tonsillectomy, and/or adenoidectomy among pediatric patients diagnosed with speech delay. The majority of patients who received procedures underwent multiple interventions, highlighting the common comorbid conditions contributing to speech delay. Ethnicity also played a role in both the prevalence of speech delay and the likelihood of undergoing at least one procedure, with Caucasian patients representing the largest proportion of patients in this category. Socioeconomic status also played a role in these outcomes, as middle- and lower-class patients had a higher predominance of undergoing procedures compared to their upper-class counterparts. 
